# Fatty acid-binding proteins in cancers

**DOI:** 10.1097/JS9.0000000000003049

**Published:** 2025-07-15

**Authors:** Long Wu, Guang-Ling Ou, Wei Zhang, Hua-Xing Ma, Xiao-Yun Li, Yun-Huan Zhen, Huan Wu, Kun Cao, Hai-Yang Li

**Affiliations:** a Department of Colorectal Cancer Center, The Affiliated Hospital of Guizhou Medical University, Guiyang 550004, Guizhou Province, China; b The Provincial Key Laboratory of Digestive System Diseases of Guizhou Province, Guiyang 550004, Guizhou Province, China; c School of Clinical Medicine, Guizhou Medical University, Guiyang 550001, China; d Key Laboratory of Hepatobiliary and Pancreatic Diseases Treatment and Bioinformatics Research, Guizhou Medical University, Guiyang 550001, China; e Department of Hepatobiliary Surgery, The Affiliated Hospital of Guizhou Medical University, Guiyang 550004, Guizhou Province, China; f Department of Infectious Diseases, The Affiliated Hospital of Guizhou Medical University, Guiyang 550004, Guizhou Province, China

**Keywords:** cancer, clinical significance, fatty acid-binding proteins, mechanisms, research progress, review

## Abstract

Fatty acid-binding proteins (FABPs) are intracellular lipid chaperones with molecular weights of approximately 14-15 kDa. By binding and transporting fatty acids and lipid-related molecules, FABPs precisely regulate metabolic pathways, signal transduction, and gene expression, playing a central role in cancer initiation and progression. The 11 identified subtypes (FABP1-FABP12; FABP11 is identical to FABP3) exhibit tissue-specific expression and influence tumor progression through metabolic reprogramming, immune microenvironment modulation, and therapy resistance. Metabolically, FABPs enhance fatty acid uptake, β-oxidation, and synthesis, meeting the high proliferative demands of tumors. In immune regulation, FABP4^+^ macrophages secrete IL-6 to suppress T cell activity, while FABP6 downregulates MHC-I molecule expression to reduce CD8^+^ T cell infiltration, fostering an immunosuppressive microenvironment. Regarding therapy resistance, FABP4 enhances mitochondrial β-oxidation to reduce apoptosis in ovarian cancer, and FABP5 promotes chemoresistance in HCC via the HIF-1α pathway. Functional heterogeneity exists among subtypes: FABP7 drives glioblastoma stem cell migration via RXRα signaling, while FABP5 exhibits context-dependent roles, promoting HCC progression but suppressing colorectal cancer (CRC) through mTOR-mediated autophagy. Clinically, FABPs serve as diagnostic biomarkers and therapeutic targets. However, challenges such as insufficient target specificity, cross-cancer heterogeneity, and normal tissue toxicity remain. Future studies should integrate multi-omics and single-cell technologies to elucidate cell-specific mechanisms and develop precise combination therapies for clinical translation.

## Introduction

Cancer remains one of the most significant global public health challenges, causing millions of deaths annually and imposing a substantial socioeconomic burden^[1]^. According to data released in 2024 by the American Cancer Society, while overall cancer mortality rates have declined since 1991, cancer incidence continues to rise^[2]^. Projections from the International Agency for Research on Cancer (IARC) suggest that by 2050, new cancer cases will reach 35 million annually^[3]^. Despite advancements in surgical, radiation, chemotherapy, and immunotherapy approaches, the complexity and heterogeneity of cancer continue to pose significant challenges to treatment outcomes. In recent years, metabolic reprogramming has emerged as a key mechanism driving tumorigenesis and progression^[4]^. Lipid metabolic reprogramming is a defining feature of cancer, enabling tumor cells to meet their energy demands and biosynthetic requirements for rapid proliferation through enhanced fatty acid uptake, oxidation, and synthesis^[5]^. Moreover, lipid metabolites affect tumor microenvironments, immune evasion, and therapeutic resistance by modulating signaling pathways and gene expression^[6]^. These metabolic aberrations not only confer survival advantages to tumors but are also closely associated with cancer aggressiveness, metastatic potential, and drug resistance^[7]^.

Fatty acid-binding proteins (FABPs), a family of intracellular lipid chaperones, play critical roles in regulating lipid metabolism, signal transduction, and gene expression by binding and transporting fatty acids and lipophilic molecules^[8]^. Recent studies have highlighted the role of FABPs in cancer, demonstrating their significant impact on tumorigenesis, progression, and prognosis through mechanisms involving metabolic reprogramming, immune microenvironment modulation, and therapeutic resistance^[9]^. Although the precise mechanisms by which FABPs influence cancer development and progression are not yet fully understood, their central role in lipid metabolism provides a novel theoretical foundation for exploring strategies in early diagnosis, prognostic evaluation, and targeted therapy. Given the growing evidence implicating FABPs in diverse oncogenic processes—ranging from metabolic reprogramming and immune microenvironment modulation to therapy resistance—a comprehensive understanding of their functions is urgently needed.

While previous reviews have explored aspects of FABPs in cancer, such as the study by Chen *et al*, which summarized their roles in metabolic disorders and cancer^[9]^, and the work by Zhang *et al*, which focused on their structural and functional characteristics^[10]^, these articles primarily address the fundamental biology of FABPs. However, a comprehensive synthesis that systematically integrates the expression patterns, molecular mechanisms, and clinical relevance of all FABP subtypes across various cancer types remains lacking. Addressing this gap, our review provides an integrated analysis encompassing molecular functions, expression profiles, and clinical implications of all FABP subtypes. We comprehensively examine their roles in a range of solid tumors, with a particular emphasis on their potential for clinical translation. Specifically, we underscore their value as diagnostic and prognostic biomarkers, as well as therapeutic targets, and highlight their multifaceted involvement in tumor metabolism, modulation of the immune microenvironment, and treatment responsiveness. Through this integrative approach, our work aims to provide a conceptual framework that supports both fundamental research and the development of targeted strategies in precision oncology. This review was conducted in accordance with the TITAN Guideline 2025^[11]^.

## Methods

We conducted a comprehensive literature search using PubMed and Web of Science databases. The primary search strategy included the terms: TS = (“FABP*” OR “Fatty acid-binding protein*”) AND (“Cancer” OR “Malignant tumor” OR “Tumor” OR “Carcinoma”). For specific FABP subtypes with multiple aliases, we expanded the search strategy accordingly. For example, for FABP1 (also known as L-FABP), the search terms included: TS = (“FABP1” OR “Fatty acid-binding protein 1” OR “L-FABP” OR “Liver fatty acid-binding protein”) AND (“Cancer” OR “Malignant tumor” OR “Tumor” OR “Carcinoma”). Articles were screened by title and abstract. Studies focusing on fatty acid-binding proteins in non-cancer contexts, or on cancer-related topics without relevance to FABPs, were excluded. Eligible articles were then reviewed in full and synthesized for inclusion in this review.

## Overview of the FABP family and its central roles

### Overview of the FABP family

Fatty acid-binding proteins (FABPs), intracellular lipid chaperones with a molecular weight of approximately 14-15 kDa, belong to the superfamily of lipid-binding proteins and exhibit significant tissue-specific expression^[12]^. So far, 11 subtypes have been identified (FABP1-FABP12, with FABP11 being identical to FABP3). Their nomenclature reflects tissue distribution, such as liver-type (FABP1), adipocyte-type (FABP4), and brain-type (FABP7). These proteins mediate the intracellular transport of hydrophobic ligands such as long-chain fatty acids, eicosanoids, and retinoic acid through a conserved β-barrel tertiary structure^[9]^.

**HIGHLIGHTS**

- FABPs drive metabolic reprogramming by enhancing fatty acid uptake, β-oxidation, and synthesis to fuel cancer proliferation, invasion, and therapy resistance across multiple tumor types.
- Immune suppression via FABP-mediated pathways: subtypes like FABP4 and FABP6 remodel the tumor microenvironment by polarizing immunosuppressive macrophages, downregulating MHC-I, and inhibiting CD8^+^ T cell infiltration.
- Functional heterogeneity among FABP isoforms: context-dependent roles are evident, such as FABP5 promoting hepatocellular carcinoma progression via HIF1α but suppressing colorectal cancer through mTOR-mediated autophagy.
- Therapeutic and diagnostic potential: FABPs serve as biomarkers for prognosis (e.g., FABP1 in gastric cancer) and targets for inhibitors (e.g., BMS309403 against FABP4), with synergy observed in combination therapies (chemotherapy/immunotherapy).
- Challenges in clinical translation: issues include cross-cancer heterogeneity, normal tissue toxicity, and insufficient specificity of inhibitors, necessitating multi-omics and single-cell approaches for precision strategies.

The core function of FABPs depends on their highly conserved ligand-binding mechanism: their internal hydrophobic cavity can specifically bind hydrophobic molecules, forming “FABP-lipid complexes” to achieve efficient transport from the cell membrane to organelles (such as mitochondria, peroxisomes, and the nucleus) or metabolic sites^[13]^. Despite low amino acid sequence homology (30%-60%), the three-dimensional structure is highly similar, consisting of 10 antiparallel β-strands forming a barrel structure that ensures high-affinity binding to hydrophobic ligands^[13,14]^. In terms of tissue distribution, FABPs exhibit significant cell-specific expression: FABP1 is primarily expressed in hepatocytes, intestinal epithelial cells, and renal tubular cells, participating in hepatic fatty acid β-oxidation and intestinal lipid absorption^[15]^. FABP4 is highly expressed in adipose tissue, with the former regulating lipid droplet dynamics in white adipocytes and the latter being involved in thermogenic metabolism in brown adipocytes^[16]^. In the nervous system, FABP7 is enriched in neuroglial cells and retinal photoreceptor cells, participating in neural lipid homeostasis and axonal development^[17]^. This spatiotemporally specific expression pattern lays the foundation for the diverse functions of FABPs in different physiological contexts.

### The central regulatory mechanism of FABPs

In maintaining energy metabolism and homeostasis, FABPs precisely regulate lipid uptake, oxidation, storage, and signaling, serving as a key link between the extracellular lipid environment and intracellular metabolic networks.

#### Coordination of fatty acid uptake and oxidation

FABPs optimize the efficiency of cellular membrane fatty acid transport and dynamically regulate downstream enzyme activity to maintain cellular energy metabolic homeostasis. For example, hepatic FABP1 can bind long-chain fatty acids (such as palmitic acid and oleic acid), promoting their rapid uptake into cells via synergy with transmembrane transport proteins (such as FATP1) and targeting them to mitochondria and peroxisomes^[18]^. During β-oxidation, FABP1 stabilizes the conformation of carnitine palmitoyltransferase 1A (CPT1A), enhancing its catalytic activity and accelerating the entry of fatty acids into mitochondria for oxidative decomposition, thereby regulating hepatic energy output^[18]^. Adipocyte FABP4 activates peroxisome proliferator-activated receptor γ (PPARγ) signaling pathways, promoting the expression of lipid droplet-related proteins (such as perilipin) and mediating triglyceride storage^[19]^. Simultaneously, its interaction with hormone-sensitive lipase (HSL) regulates lipolysis rates, maintaining a dynamic balance of free fatty acids^[19]^.

#### Metabolic reprogramming and tumor microenvironment remodeling

During tumorigenesis, abnormally activated FABPs reshape lipid metabolic patterns to meet the high energy demands of cancer cells and promote their invasive capabilities. For example, FABP5 is highly expressed in colorectal and breast cancer cells, upregulating fatty acid synthase (FASN) and sterol regulatory element-binding protein (SREBP) to drive de novo fatty acid synthesis, providing raw materials for cell membrane construction^[20]^. Simultaneously, its transport of arachidonic acid promotes the synthesis of prostaglandin E2 (PGE2), creating a pro-inflammatory microenvironment^[20]^. FABP3 enhances the degradation of GPX4, a key protein in the ferroptosis resistance pathway, by promoting the generation of arachidonic acid metabolites (such as lipoxins), thereby enhancing the resistance of tumor cells to oxidative stress-induced programmed death^[21]^. Additionally, FABP7 regulates the infiltration of immunosuppressive cell populations within glioblastomas, affecting patient prognosis and survival^[22]^. FABP7 activates nuclear receptor RXRα to drive the migration of glioblastoma stem cell-like cells (GSC), inducing tumor cell chemoresistance and recurrence^[23]^.

#### Immune cell metabolic phenotype and tumor immune microenvironment regulation

FABPs regulate the functional state and anti-tumor immune responses by remodeling the lipid metabolism characteristics of immune cells. In tumor-associated macrophages (TAMs), high expression of FABP4 promotes lipolysis, releasing free fatty acids and activating NF-κB signaling pathways, inducing the secretion of pro-inflammatory factors such as interleukin-6 (IL-6) and tumor necrosis factor-α (TNF-α), which suppress the infiltration and activation of CD8^+^ T cells^[24]^. Additionally, FABP5 in dendritic cells (DCs) regulates cholesterol ester storage, affecting the antigen presentation efficacy of MHC class II molecules and weakening adaptive immune responses^[25]^. In tumor immune evasion, FABP6 downregulates the expression of MHC class I molecules on tumor cells, reducing recognition by cytotoxic T cells and thus escaping host immune surveillance^[26]^.

Aberrant expression of FABPs is closely associated with metabolic diseases (such as obesity, type 2 diabetes, non-alcoholic fatty liver disease) and the development of tumors. In metabolic diseases, for example, circulating FABP4 levels are negatively correlated with insulin resistance and the risk of atherosclerosis^[27]^, and FABP1 inhibitors have shown potential in improving hepatic lipid deposition in animal models^[28]^. In the field of tumor therapy, small-molecule inhibitors targeting FABP4 (such as BMS-309403) can inhibit tumor angiogenesis and epithelial-mesenchymal transition (EMT), and their synergistic effects with chemotherapeutic drugs have entered preclinical research stages^[29]^. In summary, FABPs are key molecules in lipid metabolism regulation and core drivers of cancer progression, metastasis, and therapy resistance. The following sections systematically summarize the mechanisms of action and clinical significance of each FABP subtype. In addition, Table 1 summarizes FABP subtypes and their roles in cancers.

**Table 1:** Summary of FABP Subtypes and Their Roles in Cancer

FABP Name	Alias	Gene Location	Amino Acids	Tissues Expressed	Cancers Involved	Role (Promote/Inhibit)	Reference PMIDs
FABP1	Liver Type, L-FABP	2p11.2	127	Hepatocytes, intestinal epithelial cells, renal tubular cells	Hepatocellular carcinoma (HCC), gastric cancer, colorectal cancer (CRC), esophageal squamous cell carcinoma (ESCC)	Promote	27 329 821, 34 957 220, 27 884 752, 38 732 573
FABP2	Intestinal Type, I-FABP	4q26	133	Intestinal epithelial cells	CRC, breast cancer, gastric cancer, esophageal cancer, bladder cancer	Promote (Roles in CRC are inconsistent)	19 499 240, 39 242 607, 28 119 133, 34 580 877, 18 206 169
FABP3	Heart Type, H-FABP	1p35.2	133	Heart, skeletal muscle, adipocytes	Non-small cell lung cancer (NSCLC), breast cancer, CRC, esophageal cancer, gastric cancer	Promote	10 024 301, 27 323 829, 35 712 121, 38 509 965, 39 411 315
FABP4	Adipocyte Type, A-FABP, aP2, ALBP	8q21.13	132	Adipocytes, monocytes/macrophages, tumor-associated stromal cells	Breast cancer, ovarian cancer, CRC, pancreatic cancer, gastric cancer, liver cancer, esophageal cancer, thyroid cancer, lung cancer	Promote	26 260 145, 34 884 983, 32 054 768, 37 741 433, 39 411 315, 34 814 397, 35 568 702, 38 884 688, 39 353 883
FABP5	Epidermal Type, E-FABP	8q21.3	135	Epithelial cells, macrophages, stromal cells in tumor microenvironment	Liver cancer, CRC, cervical cancer, pancreatic cancer, breast cancer, prostate cancer, gastric cancer, renal cancer, lung cancer	Promote (except in CRC where it inhibits)	37 230 284, 33 128 030, 37 416 772, 32 550 890, 37 741 433, 37 741 433, 39 219 238, 39 306 229, 35 545 133
FABP6	Ileal Type, ILLBP, Gastrotropin	5q33.3	128	Intestinal tissue	CRC, glioblastoma (GBM), hepatocellular carcinoma (HCC), esophageal cancer, bladder cancer	Promote	26 579 439, 31 651 326, 34 685 761, 32 853 412, 35 216 267
FABP7	Brain Type, BLBP	6q22.31	132	Astrocytes, oligodendrocytes in central nervous system	GBM, breast cancer, melanoma, NSCLC, CRC, clear cell renal cell carcinoma (ccRCC)	Promote	24 274 717, 37 499 046, 31 324 889, 34 444 824, 35 269 427, 25 192 834
FABP8	Peripheral Nerve Type, PMP2	8q21.13	132	Peripheral nerve tissues	Hepatocellular carcinoma (HCC)	Promote (potential, via co-amplification with other FABPs)	20 421 974, 38 292 656
FABP9	Testicular Type, T-FABP, PERF15	8q21.13	132	Testis, spermatozoa acrosomes	Prostate cancer, breast cancer, esophageal squamous cell carcinoma (ESCC), HCC	Promote	15 740 583, 27 779 102, 34 536 743, 34 289 558, 38 292 656
FABP10	Intestinal/Hepatic Type, Lb-FABP	-	-	Avian liver (not detected in mammals)	Hepatocellular carcinoma (in transgenic zebrafish models)	Promote	19 540 927, 21 729 876
FABP11	FABP3	Same as FABP3					
FABP12	Prostatic Type	8q21.13	140	Prostate	Prostate cancer, hepatocellular carcinoma (HCC)	Promote	18 786 628, 33 031 638, 38 292 656

## FABP subtypes in cancers

### FABP1 (liver type)

#### Overview of FABP1

FABP1, also known as liver fatty acid-binding protein (L-FABP), is a low molecular weight soluble intracellular protein composed of 127 amino acids with a molecular weight of approximately 14 kDa, and its gene is located in the 2p11.2 region of chromosome 2^[30]^. The N-terminus of the FABP1 protein contains typical β-sheets and two short α-helices. Unlike other FABPs, FABP1 can bind two lipophilic ligands rather than a single ligand, giving it unique advantages in ligand binding capacity, functional diversity, and specificity^[31]^. FABP1 was first identified in hepatocytes and is abundantly expressed in the cytoplasm of hepatocytes and the outer membranes of mitochondria. It has also been detected in other tissues, albeit at lower levels^[15]^. FABP1 primarily regulates lipid metabolism, antioxidative stress, and is involved in the endocannabinoid system. Although new biological functions may be discovered in the future, current research remains focused on its regulatory role in lipid metabolism^[28,32]^. As a member of the FABP family, FABP1 influences lipid metabolism and thereby plays a role in the tumorigenesis and progression.

#### Roles and mechanisms of FABP1 in cancers

##### FABP1 in hepatocellular carcinoma (HCC)

In HCC, FABP1 drives tumor progression through dual mechanisms: on the one hand, it is highly expressed in tumor-associated macrophages (TAMs), promoting fatty acid oxidation via the PPARG/CD36 axis and creating an immunosuppressive microenvironment; on the other hand, FABP1 enhances the energy metabolic efficiency of hepatocellular carcinoma cells by regulating genes such as CPT1A, thereby promoting tumor metastasis^[18,33]^. In terms of targeting strategies, Orlistat inhibits FABP1 activity and synergizes with anti-PD-1 therapy to suppress HCC progression^[18]^.

##### FABP1 in gastric cancer

In gastric cancer, FABP1 expression is negatively correlated with Helicobacter pylori infection, and its progressive reduction can serve as an early diagnostic biomarker. Combined detection with TRIB3 can improve diagnostic sensitivity^[34]^.

##### FABP1 in colorectal cancer (CRC)

In metastatic CRC, FABP1 is co-expressed with lipid metabolism genes, suggesting its involvement in metabolic adaptation at metastatic sites^[35]^. Additionally, studies have shown that FABP1 expression is reduced in CRC and is associated with histologic grade, microsatellite instability (MSI), and tumor location in the right side of the colon (*P*  < 0.0001 each), and absence of BRAF V600E mutations (*P* = 0.001)^[36]^. Furthermore, low expression of FABP1 is negatively correlated with BRAF mutations, indicating its potential important role in metabolic regulation of intestinal tumors^[36]^.

##### FABP1 in esophageal squamous cell carcinoma (ESCC)

In ESCC, FABP1 is believed to be closely related to abnormal lipid metabolism. Bioinformatics analysis suggests that upregulation of FABP1 expression may promote the tumorigenesis of esophageal cancer by affecting fatty acid metabolic pathways^[37]^.

#### Clinical application of FABP1

FABP1 primarily affects lipid homeostasis in tumor metabolism. By regulating the PPARA signaling pathway, FABP1 influences the uptake of long-chain fatty acids (LCFAs) by tumor cells, thereby altering the tumor microenvironment^[38]^. Additionally, FABP1 can regulate T cell activity and affect the efficacy of immune checkpoint inhibitors (ICIs)^[38]^. The expression level of FABP1 can be used for cancer diagnosis and prognostic assessment. For example, immunohistochemical detection of FABP1 expression levels can effectively distinguish between metastatic colorectal cancer in the lung and primary pulmonary adenocarcinoma^[39]^. Furthermore, FABP1 inhibitors (such as orlistat) have been used in experimental studies and have shown potential to enhance the efficacy of immunotherapy^[18]^.

### FABP2 (intestinal type)

#### Overview of FABP2

FABP2, also known as intestinal fatty acid-binding protein (I-FABP), is a small-molecular-weight protein composed of 133 amino acids with a molecular weight of approximately 15 kDa, and its gene is located at the 4q26 region of chromosome 4^[40]^. The FABP2 protein contains 10 antiparallel β-strands (βA-J) and two short α-helices (αI, αII). Studies have found that the local unfolding of the αII helix can generate transient openings, allowing ligands to enter and exit without causing significant changes to the overall protein structure^[41]^. FABP2 is primarily expressed in intestinal epithelial cells and is closely related to the absorption of dietary fats. However, recent studies have found that abnormal expression of FABP2 is associated with the tumorigenesis and progression of various cancers and may affect the metabolic adaptability and proliferative capacity of tumor cells^[42,43]^.

#### Roles and mechanisms of FABP2 in cancers

##### FABP2 in colorectal cancer (CRC)

FABP2 gene polymorphisms and colorectal cancer susceptibility: Multiple studies have shown that single nucleotide polymorphisms (SNPs) in the FABP2 gene may affect the incidence of CRC. Research has found that the rs1799883 polymorphism in the FABP2 gene is significantly associated with CRC susceptibility, and this variant may affect fatty acid metabolism and promote CRC occurrence^[42]^. Additionally, the interaction between this polymorphism and red meat consumption may further increase CRC risk^[44]^. The role of FABP2 in the colorectal cancer microenvironment: Studies have found that FABP2 expression is regulated by the Wnt/β-catenin/PPARγ signaling pathway, which plays an important role in intestinal cell differentiation and carcinogenesis^[45]^. Furthermore, in mouse models, macrophages deficient in Smad4 enhance the FABP2/STAT6 pathway, promoting M2 macrophage polarization and accelerating CRC progression^[46]^. The role of FABP2 in colorectal cancer stem cells and differentiation: In colorectal cancer stem cell research, FABP2 has been found to co-regulate with intestinal epithelial differentiation markers (such as KLF4, CA1)^[47]^. Additionally, FABP2 shows low response to BMP4 signaling in intestinal organoid cultures, suggesting its potential impact on the differentiation capacity of colorectal cancer cells^[47]^.

##### FABP2 in breast cancer

Lipid metabolism plays an important role in the occurrence and progression of breast cancer. Studies have shown that breast cancer-associated fibroblasts (CAFs) promote lipid uptake in tumor cells through the FABP2/FABP3 pathway, thereby affecting tumor metabolism and growth. In vivo experiments, blocking FABP2 expression significantly inhibits tumor growth, suggesting that FABP2 may become an important target for metabolic regulation in breast cancer^[43]^.

##### FABP2 in gastric cancer

Recent studies have found that the tumor frontier region (Stroma AReactive Invasion Front Areas, SARIFA) in gastric cancer is associated with the abnormal expression of lipid metabolism-related genes (including FABP2, FABP4)^[48]^. This finding suggests that FABP2 may regulate tumor progression in gastric cancer through tumor-adipocyte interactions.

##### FABP2 in esophageal cancer

Studies have shown that FABP2 expression is downregulated in patients with Barrett’s esophagus and may be regulated by the gastrin signaling pathway^[49]^.

##### FABP2 in bladder cancer

FABP2 gene mutations have been identified as molecular markers of urothelial carcinoma and may aid in the early detection of bladder cancer^[50]^.

The role of FABP2 in cancer may involve multiple mechanisms, including: lipid metabolism regulation: FABP2 affects the uptake and utilization of fatty acids by tumor cells, thereby influencing tumor energy metabolism^[43]^. Signaling pathway regulation: FABP2 may influence tumor cell differentiation and proliferation through signaling pathways such as Wnt/β-catenin/PPARγ and BMP4/Notch^[45,47]^. Inflammation and immune regulation: The expression of FABP2 in macrophages may affect M2 macrophage polarization, thereby influencing the tumor microenvironment^[46]^.

#### Clinical application of FABP2

As a tumor biomarker: Changes in FABP2 expression in CRC, breast cancer, and gastric cancer suggest its potential as a biomarker for early cancer detection and prognosis assessment^[48,50]^. As a therapeutic target: Inhibiting FABP2-mediated lipid metabolism pathways may provide new therapeutic strategies for CRC and breast cancer. For example, the use of FABP2 inhibitors may reduce the dependence of tumor cells on fatty acids, thereby inhibiting tumor growth^[43]^. The role of FABP2 in various cancers is increasingly attracting attention, especially in colorectal and breast cancers, and its roles in lipid metabolism, inflammatory regulation, and the tumor microenvironment may provide new ideas for therapeutic strategies. However, the role of FABP2 may be heterogeneous across different cancers, and future research should further explore the specific mechanisms of FABP2 and evaluate its potential in clinical applications.

### FABP3 (heart type)

#### Overview of FABP3

FABP3, also known as heart fatty acid-binding protein (H-FABP), is a protein with a molecular weight of approximately 15 kDa. Its gene is located in the 1p35.2 region of human chromosome 1 and encodes 133 amino acids. FABP3 is primarily expressed in the cytoplasm of tissues such as the heart, skeletal muscle, and adipocytes, participates in lipid transport and metabolism and provides energy for myocardial cells^[51]^. Research has confirmed that FABP11 is identical to FABP3^[52]^. FABP3 can bind long-chain fatty acids (LCFAs) and transport them to specific intracellular sites for energy production and metabolic regulation^[53]^. FABP3 plays a significant role in the pathogenesis of cardiovascular and metabolic diseases. Studies have shown that elevated FABP3 levels are closely associated with an increased risk of metabolic diseases such as obesity, diabetes, and hypertension^[54–58]^. Additionally, FABP3 is involved in the pathological processes of cardiovascular diseases such as myocardial ischemia-reperfusion injury, myocardial hypertrophy, and heart failure^[59,60]^. In recent years, dysregulated expression of FABP3 and its potential mechanisms in various cancers have attracted the attention of researchers.

#### Roles and mechanisms of FABP3 in cancers

##### FABP3 in non-small cell lung cancer (NSCLC)

Studies have shown that high expression of FABP3 and FABP4 is associated with poor prognosis in NSCLC patients. The expression level of FABP3 in cancer tissues is significantly higher than that in normal tissues and is positively correlated with TNM staging^[61]^.

##### FABP3 in breast cancer

In the tumor-adipose microenvironment (TAME) of breast cancer, the expression of FABP3 in lipid-associated macrophages (LAMs) is elevated. These cells are enriched at the tumor-adipose interface and exhibit pro-tumorigenic effects. Additionally, FABP3 may influence breast cancer progression through lipid metabolism and immune regulation^[62]^.

##### FABP3 in colorectal cancer (CRC)

FABP3 has been identified as an independent prognostic factor for poor outcomes in patients with colon adenocarcinoma (COAD). Bioinformatics analysis based on transcriptomic data showed that FABP3 is associated with the PPAR signaling pathway and may play a role in metabolic reprogramming in CRC^[63]^.

##### FABP3 in esophageal cancer

FABP3 has been included in a multi-gene scoring model for predicting the prognosis of esophageal cancer patients. This model performed well in survival analysis, suggesting that FABP3 may serve as a potential biomarker for esophageal cancer^[64]^.

##### FABP3 in gastric cancer

In gastric cancer patients with cancer-associated cachexia (CAC), plasma levels of FABP3 are significantly elevated, suggesting its potential role in cancer-related muscle wasting^[65]^.

FABP3 is primarily involved in the transport and metabolic regulation of intracellular fatty acids and is highly expressed in cardiac and skeletal muscle tissues. Its abnormal expression is closely associated with mitochondrial dysfunction, oxidative stress, and cell apoptosis^[66]^. The mechanisms of FABP3 in cancer include the following: mitochondrial dysfunction and cell apoptosis: Overexpression of FABP3 in embryonic cancer cells leads to reduced mitochondrial membrane potential, decreased ATP synthesis, and increased oxidative stress, ultimately inducing cell apoptosis^[53]^. Lipid metabolism and cancer cell adaptation: Research in breast cancer has found that FABP3 expression is negatively correlated with lipid transport protein FATP1, suggesting that FABP3 may play a regulatory role in metabolic adaptation between cancer cells and stromal cells^[43]^. Tumor immune microenvironment: FABP3 is involved in a copper death-related immune signature and plays a role in prognosis prediction for gastric cancer patients, potentially influencing disease progression through regulation of the tumor immune microenvironment^[67]^.

#### Clinical application of FABP3

Abnormal expression of FABP3 is associated with poor prognosis in various cancers, suggesting its potential as a biomarker for cancer diagnosis and prognosis assessment. Inhibitors targeting FABP3 may become novel strategies for cancer therapy. For example, some studies have commenced to evaluate the safety and therapeutic potential of FABP3 inhibitors in cancers^[68]^. In breast cancer immunotherapy models, inhibiting FABP3-enriched LAMs enhances the efficacy of anti-PD-1 therapy, suggesting that FABP3 may serve as a co-target for immunotherapy^[62]^. FABP3 plays a crucial role in the occurrence and development of various cancers, through mechanisms involving mitochondrial function, lipid metabolism, and tumor immune regulation. Future research exploring the molecular mechanisms of FABP3 and its potential as a therapeutic target may contribute to the development of more effective cancer diagnosis and treatment strategies.

### FABP4 (adipocyte type)

#### Overview of FABP4

FABP4, also known as adipocyte-type fatty acid-binding protein (A-FABP, aP2, ALBP), is an intracellular lipid-binding protein with a molecular weight of approximately 15 kDa and belongs to the FABP family. The human FABP4 gene is located in the 8q21.13 region of chromosome 8, and the FABP4 protein encoded by it consists of 132 amino acids. Its structure includes 10 β-sheets and 2 α-helices, forming a hydrophobic ligand-binding cavity that specifically binds long-chain fatty acids and eicosanoids^[16]^. FABP4 is primarily expressed in adipocytes, monocytes/macrophages, and tumor-associated stromal cells, regulating fatty acid uptake, transport, and metabolism, and participating in energy homeostasis, inflammatory responses, and cellular signal transduction.

#### Roles and mechanisms of FABP4 in cancer

##### FABP4 in breast cancer

In the tumor-adipose microenvironment, FABP4 is highly expressed and drives breast cancer progression through metabolic reprogramming, immune suppression, and metastatic adaptation. Serum FABP4 levels are negatively correlated with overall survival in patients^[69]^, and its overexpression promotes lipid uptake via CD36 synergy, activating the STAT3 pathway to induce epithelial-mesenchymal transition (EMT) and stemness in triple-negative breast cancer (TNBC)^[69,70]^. In obese TNBC patients, FABP4 upregulates ANGPTL4 and CD36, enhancing tumor angiogenesis and chemoresistance^[71]^. FABP4^+^ lipid-associated macrophages (LAMs) further suppress antitumor immunity by secreting IL-6 and TGF-β, which inhibit CD8^+^ T cell activity^[62]^. Mechanistically, FABP4 mediates paracrine lipid transfer from tumor-associated adipocytes to cancer cells, fueling mitochondrial β-oxidation and upregulating stemness markers (e.g., ALDH1A1), thereby sustaining aggressive phenotypes^[72]^.

##### FABP4 in ovarian cancer

High expression of FABP4 is significantly associated with peritoneal metastasis, chemoresistance, and shortened patient survival in ovarian cancer. Ovarian cancer cells take up fatty acids released by adipocytes through FABP4, promoting their survival and metastasis. FABP4 inhibits 5-hydroxymethylcytosine accumulation and upregulates the expression of pro-metastatic genes (e.g., SNAI1, TWIST1)^[29]^. FABP4 reduces apoptosis in ovarian cancer by enhancing mitochondrial β-oxidation^[73]^.

##### FABP4 in colorectal cancer (CRC)

High expression of FABP4 is associated with liver metastasis, chemoresistance, and poor patient prognosis in CRC^[74]^. FABP4 promotes tumor cell proliferation and EMT through the PPARγ/β-catenin axis^[75]^. FABP4 regulates the expression of GPX4 and SLC7A11, inhibiting ferroptosis and enhancing oxaliplatin resistance^[76]^. FABP4 promotes glycolysis and stemness through the ROS/ERK/mTOR pathway^[77]^. In CRC, FABP4, together with CD36 and SCD, mediates fatty acid uptake in tumor cells, promoting liver and lung metastasis^[78]^. High FABP4 expression is associated with M2 polarization of tumor-associated macrophages (TAMs), enhancing the immunosuppressive microenvironment^[79]^. High FABP4 expression is associated with lymph node metastasis and poor prognosis, and a 12-gene model composed of FABP4 and other genes can effectively predict survival^[80]^. In metastatic CRC, FABP4 upregulates UCP2 to enhance mitochondrial oxidative phosphorylation (OXPHOS), reducing oxaliplatin sensitivity^[81]^. FABP4 activates NRF2 and ERK1/2 signaling, reducing the efficacy of EGFR inhibitors (e.g., cetuximab)^[82]^. In tumors with high FABP4 expression, PD-L1 expression is upregulated and CD8 + T cell infiltration is reduced, indicating a lower response rate to immune checkpoint inhibitors (ICIs)^[83]^.

##### FABP4 in pancreatic cancer

FABP4 is highly expressed in obesity-associated pancreatic cancer, promoting tumor progression and immunosuppression^[84]^. FABP4 mediates the NLRP3/IL-1β pathway, inducing pyroptosis in macrophages and promoting tumor metastasis^[84]^. FABP4 synergizes with SCD1 to regulate lipid metabolism and resistance to ferroptosis^[76]^.

##### FABP4 in gastric cancer

FABP4 is highly expressed in gastric cancer tissues and is associated with tumor invasion and cachexia^[65]^. Enhanced fatty acid oxidation: FABP4 promotes the uptake of free fatty acids by tumor cells, maintaining mitochondrial function^[85]^. FABP4 upregulates PD-L1 expression through the STAT3 pathway, inhibiting T-cell immunity^[86]^.

##### FABP4 in liver cancer

FABP4 promotes lipid accumulation and proliferation in liver cancer cells through the PPARγ pathway^[63]^. FABP4 expression is positively correlated with the degree of steatosis in liver cancer and is associated with an increased risk of postoperative recurrence^[87]^. FABP4 upregulates NRF2 and HO-1, reducing ROS accumulation and protecting liver cancer cells from oxidative damage^[88]^.

##### FABP4 in esophageal cancer

FABP4 is associated with epithelial-mesenchymal transition (EMT) and angiogenesis in esophageal cancer, contributing to tumor invasion^[64]^. In esophageal cancer, FABP4 inhibits AMPK phosphorylation, activates mTOR signaling, and promotes tumor cell proliferation^[89]^.

##### FABP4 in other cancers

*Thyroid cancer:* FABP4 mRNA and protein levels are reduced in PTC and FTC and may serve as potential indicators of thyroid cancer development^[90]^. FABP4 has good predictive value for the prognosis of thyroid cancer patients and may serve as a clinical biomarker for diagnosis and prognosis^[91]^.

*Lung cancer:* FABP4 + macrophages support tumor growth through lipid metabolism and are associated with resistance to anti-PD1 therapy^[92]^.

#### Clinical application of FABP4

The expression level of FABP4 can serve as an independent prognostic indicator for cancers such as breast and ovarian cancer. FABP4 inhibitors such as BMS309403 significantly inhibit tumor growth and enhance chemosensitivity in ovarian cancer models^[29]^. Targeting FABP4 can reshape the tumor immune microenvironment and enhance the efficacy of PD-1 inhibitors^[62]^. Therapeutic strategies targeting FABP4 are under exploration: small-molecule inhibitors: such as BMS309403, which competitively binds to the fatty acid binding site of FABP4, inhibiting its pro-metastatic effects^[81]^. Novel pyridazinone compounds (14e) exhibit high inhibitory activity against FABP4(IC50 = 1.57 μM)^[93]^. Combination therapy: FABP4 inhibitors used in combination with chemotherapy (5FU) or immunotherapy (PD-1 antibodies) significantly enhance antitumor effects^[81]^. Metabolic intervention: Inhibiting fatty acid uptake (e.g., CD36 antibodies) or lipid droplet formation (e.g., SCD inhibitors) can synergistically inhibit FABP4 function^[78]^. Clinical application of FABP4 also faces some challenges, such as the need to further clarify the functional heterogeneity of FABP4 across different cancers. The specificity and in vivo stability of existing inhibitors need optimization^[94]^. Future research needs to further clarify the cell type-specific effects of FABP4 and develop tissue-selective inhibitors. As a lipid metabolism hub molecule, FABP4 plays a central role in cancer progression by regulating the tumor microenvironment, immune evasion, and metabolic adaptation. Its potential as a prognostic biomarker and therapeutic target has been widely validated, but clinical translation still needs to overcome challenges related to target specificity, drug resistance mechanisms, and cross-cancer heterogeneity^[71,81]^.

### FABP5 (epidermal type)

#### Overview of FABP5

Fatty acid-binding protein 5 (FABP5), also known as epidermal fatty acid-binding protein (E-FABP), is an intracellular lipid chaperone with a molecular weight of approximately 15 kDa. It is responsible for transporting fatty acids to nuclear receptors (e.g., PPARγ) and regulating lipid metabolism, cell proliferation, and inflammatory signaling pathways^[95]^. Its gene is located at human chromosome 8q21.3 and encodes 135 amino acids. FABP5 is primarily expressed in epithelial cells, macrophages, and stromal cells within the tumor microenvironment. FABP5 is abnormally overexpressed in various cancers, driving metabolic reprogramming, immune suppression, and metastasis through the activation of pro-tumorigenic transcription factors, and is significantly associated with poor prognosis^[96,97]^.

#### Roles and mechanisms of FABP5 in cancers

##### FABP5 in hepatocellular carcinoma (HCC)

FABP5 is highly expressed in hepatocellular carcinoma (HCC) and promotes tumor progression through multiple mechanisms. FABP5 binds to HIF-1α, promoting its nuclear translocation, thereby enhancing fatty acid metabolism and tumor cell survival^[96]^. FABP5^+^ tumor-associated macrophages (TAMs) secrete immunosuppressive factors (e.g., IL-10), suppressing CD8^+^ T cell activity and creating an immunosuppressive microenvironment^[98]^. FABP5 inhibition induces ferroptosis via lipid peroxidation and synergizes with chemotherapy to enhance therapeutic efficacy^[99]^. FABP5 overexpression is significantly associated with poor prognosis in HCC patients, indicating its potential as a therapeutic target^[99]^.

##### FABP5 in colorectal cancer (CRC)

In colorectal cancer, FABP5 promotes tumor progression by regulating lipid metabolism and cell proliferation-related signaling pathways. FABP5 downregulates fatty acid synthase (FASN), activating mTOR-mediated autophagy and inhibiting CRC cell proliferation and metastasis^[100]^. Hypomethylation of the FABP5 promoter forms a positive feedback loop with NF-κB activation, promoting CRC invasion^[101]^. FABP5 interacts with PPARγ to enhance its transcriptional activity, thereby promoting CRC cell proliferation and invasion^[101]^. High FABP5 expression is associated with poor prognosis in CRC patients^[101]^.

##### FABP5 in cervical cancer

FABP5 is highly expressed in cervical cancer, promoting lymph node metastasis and immunosuppression. FABP5 activates NF-κB by promoting fatty acid metabolism, inducing epithelial-mesenchymal transition (EMT) and lymphangiogenesis, thereby facilitating cervical cancer metastasis^[102]^. FABP5 is negatively regulated by miR-144-3p and promotes metastasis in hypoxic microenvironments^[102]^. FABP5 inhibitors (e.g., orlistat) significantly suppress tumor growth and metastasis, and may enhance the efficacy of immunotherapy^[102]^. FABP5 may serve as a biomarker for pelvic lymph node metastasis in cervical cancer^[103]^. Resveratrol (RSV) directly interacts with FABP5, inhibiting fatty acid transport to the nucleus and downstream matrix metalloproteinases (MMP2 and MMP9), thereby suppressing cervical cancer metastasis^[104]^.

##### FABP5 in pancreatic neuroendocrine neoplasms (pNENs)

FABP5 promotes tumor progression in pancreatic neuroendocrine neoplasms by regulating lipid metabolism and the mTOR signaling pathway. High FABP5 expression is significantly associated with poor prognosis in pNEN patients, and FABP5 interacts with the PPARγ signaling pathway to enhance tumor cell proliferation and invasion^[105]^.

##### FABP5 in breast cancer

FABP5 is highly expressed in breast cancer, particularly in triple-negative breast cancer (TNBC), and is associated with poor prognosis, high tumor grade, and aggressiveness^[106–108]^. FABP5 promotes breast cancer progression by regulating lipid metabolism and interacting with lipid-associated macrophages (LAMs) in the tumor microenvironment^[107]^. FABP5 activates signaling pathways such as PPARβ/δ, EGFR, and CaMKII, enhancing tumor cell proliferation, invasion, and chemoresistance^[109,110]^. FABP5 inhibitors such as SBFI-26 suppress tumor cell proliferation and invasion by inducing ferroptosis or enhancing chemotherapeutic sensitivity, showing therapeutic potential^[111,112]^.

##### FABP5 in prostate cancer

FABP5 promotes prostate cancer proliferation and invasion by regulating lipid metabolism and the PPARγ signaling pathway. High FABP5 expression is significantly associated with poor prognosis in prostate cancer patients, and FABP5 inhibitors (e.g., SBFI-26) suppress tumor cell proliferation and invasion^[113]^. FABP5 interacts with androgen receptors (AR) to promote castration-resistant prostate cancer (CRPC) progression^[113]^.

##### FABP5 in melanoma

FABP5 exhibits diverse functions in melanoma, with its expression levels closely associated with tumor progression, metastasis, and poor prognosis. Studies have demonstrated that FABP5 is upregulated in melanoma and linked to tumor cell proliferation, metabolism, and malignant progression^[114]^. In terms of prognosis, high expression of FABP5 correlates with shorter overall survival (OS) and poorer progression-free survival (PFS) in melanoma patients^[115]^. Additionally, FABP5 is closely associated with tumor-related signaling pathways, such as PPAR-γ, NF-κB, and IL-6/STAT3, and may promote tumor progression and metastasis through these pathways^[116]^. Furthermore, FABP5 has been identified as associated with monocytic subsets in metastatic melanoma, potentially enhancing tumor migration and survival^[117,118]^.

##### FABP5 in other cancers

*Lung cancer:* In lung cancer, CCAT1/FABP5 promotes tumor progression through mediating fatty acid metabolism and stabilizing PI3K/AKT/mTOR signaling^[119]^.

*Gastric cancer:* FABP5 overexpression is closely associated with poor prognosis in gastric cancer. Ectopic expression of FABP5 promotes gastric cancer proliferation, invasion, migration, and carcinogenicity. Additionally, RNA-seq analysis revealed that FABP5 activates immune-related pathways, including cytokine-cytokine receptor interaction, interleukin-17 signaling, and tumor necrosis factor (TNF) signaling. These findings highlight the potential importance of combining FABP5-targeted therapies with immunotherapy for gastric cancer treatment^[120]^.

*Bladder Cancer* (BCa): Research indicates that FABP5 can directly interact with lncDBET, thereby activating the PPAR signaling pathway. This interaction enhances lipid metabolism in cancer cells, thereby promoting the malignant progression of BCa both in vivo and in vitro^[121]^.

*Renal Cell Carcinoma (RCC):* FABP5 is upregulated in RCC tissues and cell lines, and it is positively correlated with the progression of RCC. Depletion of FABP5 inhibits the proliferation, colony formation, and migration of RCC cells. Mechanistically, FABP5 depletion significantly downregulates MMP9 and the transcription factor Snail1, while upregulating E-cadherin and downregulating N-cadherin and Vimentin. These changes suppress epithelial-mesenchymal transition (EMT) in the ACHN cell line^[122]^.

*Ovarian Cancer:* In ovarian cancer, FABP5 interacts with TAGLN2, promoting TAGLN2 cell surface localization and its role in activated CD8 + T cells^[97]^.

#### Clinical applications of FABP5

FABP5 demonstrates significant prognostic value and therapeutic potential across multiple cancers. High FABP5 expression is associated with poor prognosis in various cancers, indicating its potential as a biomarker for early diagnosis and prognostic assessment^[96,101,105,108,113,119,120,122–125]^. FABP5 inhibitors (e.g., SBFI-26) show significant antitumor effects in multiple cancer models, suggesting their potential as novel therapeutic agents^[105,113,124]^. However, clinical translation faces challenges, including FABP5’s physiological roles in normal tissues (e.g., lipid metabolism regulation), tissue-specific regulatory mechanisms, and tumor microenvironment effects (e.g., stromal fibrosis). Future studies should clarify FABP5’s tissue-specific mechanisms, develop efficient targeted drugs, and explore personalized combination therapies. Potential strategies include developing antibodies or nanomedicines targeting secreted FABP5, investigating synergistic effects of FABP5 inhibitors with immune checkpoint inhibitors or chemotherapy, and using multi-omics approaches to dissect dynamic FABP5 regulatory networks in the tumor microenvironment.

### FABP6 (ileal type)

#### Overview of FABP6

FABP6, a member of the fatty acid-binding protein family, is also known as ileal lipid-binding protein (ILBP), gastrotropin, with a molecular weight of 15 kDa. Its gene is located on human chromosome 5 at position 5q33.3, and it has multiple transcript variants encoding 128 amino acids. It primarily participates in intracellular lipid transport and metabolic regulation. By binding to bile acids and fatty acids, it regulates metabolic pathways and signal transduction, playing an important role in digestive system cancers^[26,126]^.

#### Roles and mechanisms of FABP6 in cancers

##### FABP6 in colorectal cancer (CRC)

FABP6 is significantly overexpressed in colorectal cancer tissues and serum, showing a clear difference compared with normal tissues, and can serve as a potential diagnostic biomarker^[126,127]^. High expression of FABP6 is associated with poor prognosis in CRC patients. It inhibits CD8+ T cell infiltration by downregulating MHC-I molecules expression and immune-related chemokine secretion, promoting immune evasion^[26,128]^. FABP6 promotes CRC cell proliferation and metastasis by regulating bile acid metabolism and insulin-like growth factor (IGF) signaling pathway^[129,130]^. FABP6 promotes oxaliplatin resistance in CRC through KLF5-dependent transcription, and its high expression is associated with lipid droplet formation and Wnt signaling pathway activation^[131]^. Knockdown of FABP6 enhances MHC-I expression and recruits CD8^+^ T cells, indicating its immunomodulatory role^[26]^.

##### FABP6 in glioblastoma (GBM)

FABP6 regulates tumor progression through ERK, JNK, and NF-κB pathways, and combined with temozolomide therapy can enhance therapeutic efficacy^[132]^. Inhibition of FABP6 reduces glioma cell migration and invasion, reduces MMP-2 and VEGF expression, and inhibits angiogenesis^[132]^.

##### FABP6 in hepatocellular carcinoma (HCC)

FABP6 is highly expressed in hepatocellular carcinoma and is significantly associated with shortened overall survival in patients. It affects the tumor microenvironment by regulating metabolic and immune-related pathways (e.g., mTOR, Wnt)^[133,134]^. High expression of FABP6 is associated with an immunosuppressive microenvironment, inhibiting B cell memory and dendritic cell function^[135]^. In hepatocellular carcinoma, FABP6 is associated with bile acid metabolic reprogramming and can be included in a multi-gene model to predict sorafenib resistance^[136]^.

##### FABP6 in other cancers

*Esophageal Cancer*: FABP6^+^ tumor cells interact with T cells through the MIF pathway, promoting immune suppression^[137]^.

*Bladder Cancer:* FABP6 promotes tumor proliferation and migration by regulating autophagy and cell cycle proteins (CDK2/4)^[130]^.

The role of FABP6 in cancer involves multiple molecular mechanisms: metabolic regulation: FABP6 affects tumor cell energy supply through bile acid transport and fatty acid metabolism^[126,129]^. Immune evasion: Downregulation of MHC-I expression inhibits CD8^+^ T cell infiltration and reduces immunogenicity^[26]^. Signaling pathways: Activation of ERK, JNK, NF-κB, and other pathways promotes invasion and angiogenesis^[132]^.

#### Clinical application of FABP6

Multiple studies have confirmed that FABP6 is an independent prognostic risk factor for CRC, HCC, and other cancers^[127,133]^. Combined detection of FABP6 with CEA can improve the sensitivity of colorectal cancer diagnosis^[127]^. Inhibition of FABP6 can enhance the sensitivity of chemotherapeutic drugs (e.g., oxaliplatin) and reverse immunotherapy resistance^[131,132]^. FABP6 plays a pro-tumorigenic role in various cancers through metabolic reprogramming, immune regulation, and activation of signaling pathways, and is a potential diagnostic biomarker and therapeutic target. Future research needs to further validate its clinical translation value and explore precision therapeutic strategies targeting FABP6.

### FABP7 (brain type)

#### Overview of FABP7

FABP7, also known as brain fatty acid-binding protein (BLBP), has a molecular weight of approximately 15 kDa and is primarily distributed in astrocytes and oligodendrocytes of the central nervous system. The FABP7 gene is located in the 6q24.31 region of human chromosome 6 and encodes 132 amino acids. FABP7 is an intracellular chaperone for various omega-3 fatty acids and is known as a neural stem cell marker^[17]^. FABP7 is responsible for the uptake, transport, and metabolic regulation of fatty acids. In recent years, its biological functions and clinical implications in various cancers have been widely studied.

#### Roles and mechanisms of FABP7 in cancer

##### FABP7 in glioblastoma (GBM)

In GBM, FABP7 increases the accumulation of monounsaturated fatty acids (MUFA) and triglycerides, inhibiting lipid peroxidation and helping cancer cells resist ferroptosis^[138]^. FABP7 is highly expressed in glioblastoma tumor stem cells (GSCs) and drives the migration and infiltration of glioblastoma cells through the RXRα signaling pathway. Knockdown of FABP7 reduces the migration and infiltration of tumor stem cells and enhances radiotherapy sensitivity^[23]^. FABP7 drives lipid droplet formation and radioresistance in glioblastoma stem cells through the RXRα signaling pathway^[139]^. FABP7 epigenetically reprograms to suppress immune-related genes (e.g., LPCAT3) while upregulating ferroptosis-resistant genes (e.g., BMAL1), helping glioblastoma cells evade immune clearance^[138]^. FABP7 expression is significantly associated with reduced immune cell infiltration and shortened survival in low-grade glioma (LGG) patients^[22]^. FABP7 inhibitor MF6 can suppress glioblastoma, while supplementation of DHA (a FABP7 ligand) can inhibit glioma migration^[140]^.

##### FABP7 in breast cancer

In breast cancer, FABP7 promotes the formation of an immunosuppressive microenvironment by regulating angiogenesis-related genes (VEGFA/P4HA1)^[22]^. In brain metastasis of breast cancer, FABP7 mediates a glycolytic phenotype to adapt to the brain microenvironment^[141]^. FABP7 can modulate 13-HODE-mediated linoleic acid-induced cell death in triple-negative breast cancer cells^[142]^. Patients with high FABP7 expression in breast cancer have poor responses to neoadjuvant chemotherapy (e.g., paclitaxel and anthracyclines), but its expression level can predict the efficacy of anti-angiogenic drugs (e.g., apatinib)^[143]^.

##### FABP7 in other cancers

*Melanoma:* In melanoma, FABP7 knockdown reduces the proliferation and migration of SK-MEL-23 cells, cyclin D1 expression, and Wnt/β-catenin activity. Ligand-bound FABP7 can drive melanoma cell proliferation by modulating Wnt/β-catenin signaling^[144]^.

*Non-small cell lung cancer (NSCLC):* FABP7 enhances the metastatic capability of NSCLC through activation of the Wnt/β-catenin pathway^[145]^.

*Colorectal cancer:* High expression of FABP7 activates the MEK/ERK signaling pathway in colorectal cancer, inhibits cell apoptosis, and promotes cell cycle progression^[146]^.

*Clear cell renal cell carcinoma (ccRCC):* High expression of FABP7 indicates advanced clinical staging and poorer survival^[147]^.

#### Clinical application of FABP7

A comparative analysis of breast cancer subtypes based on cancer cell heterogeneity reveals that FABP7 may act as a tumor suppressor, holding significant clinical implications for diagnosis, prognosis, and targeted therapy in breast cancer patients^[148]^. Additionally, studies indicate that FABP7 overexpression is significantly associated with the life expectancy and overall survival (OS) of TNBC patients^[149]^. Furthermore, FABP7 expression is significantly linked to reduced immune cell infiltration and shorter survival in low-grade glioma (LGG) patients^[22]^. FABP7 may be used as a novel diagnostic biomarker and a potential therapeutic target for colon cancer^[146]^. FABP7 overexpression indicates advanced clinical staging and shorter survival^[147]^. Additionally, FABP7 shows promise as a therapeutic target. For example, in glioblastoma, knockdown of FABP7 enhances radiotherapy sensitivity^[23]^. Small-molecule inhibitors targeting FABP7 (e.g., ART26.12) significantly inhibit tumor growth and prolong survival in animal models^[68]^. The combination of FABP7 with immune checkpoint inhibitors such as PD-1/PD-L1 can reverse tumor immune evasion and enhance therapeutic efficacy^[138]^.

Although most studies indicate that FABP7 has oncogenic effects, some studies suggest that it may have protective effects. For example, nuclear localization of FABP7 in breast cancer is associated with longer disease-free survival^[150]^. This discrepancy may arise from functional differences of FABP7 in different subcellular localizations or tumor microenvironments. FABP7 plays a key role in various cancers by regulating lipid metabolism, tumor stem cell properties, and the immune microenvironment. Its potential as a prognostic biomarker and therapeutic target has been preliminarily validated, but further research is needed to clarify its functional heterogeneity and clinical translation strategies.

### FABP8 (peripheral nerve type)

#### Overview of FABP8

FABP8, also known as peripheral nerve-type FABP, peripheral myelin protein 2 (PMP2), is an alkaline cytoplasmic protein with a molecular weight of 14.5 kDa. Its gene is located in the 8q21.13 region of human chromosome 8 and encodes 132 amino acids^[151,152]^. FABP8 is primarily studied for its role in the nervous system, particularly in myelination, lipid metabolism, and nerve regeneration^[153–155]^. However, its direct role in cancer has been less researched to date.

#### Roles and mechanisms of FABP8 in cancer

##### FABP8 in hepatocellular carcinoma

In hepatocellular carcinoma (HCC), co-amplification of FABP8 with other FABP members (e.g., FABP5) may promote tumor progression by regulating lipid metabolism, although the specific mechanism remains unclear^[156]^. FABP8 often participates in lipid metabolism regulation together with other FABP family members. For example, FABP5 is significantly overexpressed in liver cancer and is closely associated with cell cycle regulatory genes (CDK1, CDK4) and apoptosis inhibitory genes (BIRC5)^[156]^. FABP8 may influence tumor cell proliferation or survival through similar pathways, although direct evidence is currently lacking.

##### FABP8 in melanoma

FABP8 (PMP2) is a direct target of SOX10, a transcription factor critical for melanocyte, and glial cell differentiation. Studies indicate that PMP2 expression enhances the invasive capacity of melanoma cells. However, PMP2 protein expression is only detected in certain melanoma cell lines, which may reflect the heterogeneity of melanoma^[157]^.

##### FABP8 in ovarian cancer

FABP8 (PMP2) has been identified as a potential blood-based biomarker for ovarian cancer. Metabolomic and proteomic analyses suggest that PMP2 is associated with cancer plasticity and signaling pathways, potentially offering a new target for early diagnosis^[158]^.

##### FABP8 in Schwannoma

FABP8 (PMP2) exhibits a specific expression pattern in schwannomas. Research indicates that SOX10 insertion/deletion mutations impair its transcriptional activation of glial differentiation and myelination gene programs, with PMP2 being part of these programs. Aberrant PMP2 expression may contribute to schwannoma development^[159]^.

In pan-cancer analyses, FABP8 forms a gene cluster with FABP4, FABP5, FABP9, and FABP12, which frequently undergo co-amplification in various cancers^[156]^. This amplification may lead to FABP8 overexpression, influencing lipid metabolism and signaling pathways in tumor cells. As a fatty acid-binding protein, FABP8 regulates membrane lipid distribution (e.g., sphingolipid transbilayer movement) and depends on PI(4,5)P2 signaling to affect membrane dynamics^[160]^. These functions may indirectly impact cancer progression by altering membrane physical properties or signal transduction. Additionally, FABP8 enhances mitochondrial ATP production, potentially supporting the high metabolic demands of tumor cells, though its specific metabolic role in cancer remains under investigation^[153]^.

#### Clinical applications of FABP8

The role of FABP8 in cancer is still in its early stages of exploration, with evidence primarily based on its co-amplification with FABP family members and its potential links to lipid metabolism. Emerging evidence suggests that FABP8 may serve as a biomarker or therapeutic target in certain cancers, such as ovarian cancer^[158]^. Future research should clarify FABP8 expression patterns in tumor cells and its effects on lipid metabolism, inflammation, and energy homeostasis. If FABP8 proves critical in specific cancers, its inhibitors could become novel therapeutic strategies, similar to those explored for FABP5 in hepatocellular carcinoma.

### FABP9 (testicular type)

#### Overview of FABP9

FABP9, also known as testicular-type fatty acid-binding protein (Testis Fatty Acid-Binding Protein, T-FABP, PERF15), is a member of the fatty acid-binding protein family with a molecular weight of 15 kDa. Its gene is located in the 8q21.13 region of human chromosome 8 and encodes 132 amino acids. FABP9 is highly expressed in the inner membrane of sperm acrosomes and is associated with germ cell apoptosis^[161]^. Recently, studies have found that FABP9 is abnormally expressed in various cancers and is closely related to tumor progression, invasion, and patient prognosis.

#### Roles and mechanisms of FABP9 in cancer

##### FABP9 in prostate cancer

FABP9 is significantly overexpressed in malignant prostate cancer cell lines (e.g., PC-3, PC3-M) and prostate cancer tissues. Its expression level is significantly associated with shortened patient survival, elevated Gleason scores, and enhanced androgen receptor (AR) activity^[162]^. Inhibition of FABP9 reduces the invasiveness of highly invasive prostate cancer cells (PC3-M), suggesting that it promotes tumor invasion through lipid metabolism regulation^[162]^.

##### FABP9 in breast cancer

FABP9 is included in an immune gene-based prognostic model for breast cancer, and its high expression is significantly associated with reduced overall survival^[163]^. FABP9 mRNA levels are significantly higher in tumor tissues than in normal tissues, suggesting that it may promote breast cancer progression through lipid metabolism or immune microenvironment regulation^[163]^.

##### FABP9 in esophageal squamous cell carcinoma (ESCC)

FABP9 has been identified as one of seven immune-related genes associated with ESCC patient survival and can serve as an independent prognostic risk gene for esophageal cancer. Its high expression is associated with increased CD4^+^ T lymphocyte infiltration, which may affect the tumor immune microenvironment^[164]^.

##### FABP9 in hepatocellular carcinoma and other cancers

In hepatocellular carcinoma (HCC), FABP9 co-amplifies with other FABP family members (e.g., FABP4, FABP5), which may synergistically promote lipid metabolic reprogramming and tumor progression. Pan-cancer analysis has found that FABP9 is widely involved in tumor occurrence through key pathways such as G2/M checkpoint regulation and TP53 signaling pathway, co-expressing with cell cycle and apoptosis-related genes (e.g., CDK1, PLK1)^[156]^.

The mechanisms of FABP9 in cancer may include: lipid metabolism and invasion: FABP9 promotes tumor cell energy supply and membrane structure remodeling by binding to fatty acids, enhancing invasive capacity^[162]^. Immune regulation: In ESCC, FABP9 may affect anti-tumor immune responses by regulating CD4 + T cell infiltration^[164]^. Epigenetic regulation: FABP9 is associated with histone modification (H3K4me3) during spermatogenesis, suggesting that it may regulate gene expression through epigenetic mechanisms in tumors^[165]^.

#### Clinical application of FABP9

FABP9 can serve as a tumor prognostic biomarker: High expression of FABP9 is significantly associated with poor prognosis in various cancers, including prostate cancer, breast cancer, and ESCC, and has potential clinical diagnostic value^[162–164]^. FABP9 as a potential therapeutic target for tumors: Inhibiting FABP9 can reduce tumor cell invasiveness, especially in FABP9-overexpressing liver cancer, and targeted intervention may become a new strategy^[156]^. However, there are also controversies and challenges: tissue specificity: FABP9 is highly expressed in the reproductive system (e.g., testes) with clear functions, but whether its expression in tumors is regulated by reproductive-related signals remains to be verified^[166,167]^. Mechanistic diversity: The mechanisms of FABP9 may vary across different cancers, and further research is needed on its upstream and downstream pathways and interacting proteins. In summary, FABP9 promotes cancer through lipid metabolism, immune microenvironment, and epigenetic modification, and its high expression is significantly associated with poor prognosis. Future research needs to further clarify its tissue-specific mechanisms and explore precision therapeutic strategies targeting FABP9.

### FABP10 (intestinal/hepatic type)

#### Overview of FABP10

FABP10, also known as liver basic fatty acid-binding protein (Lb-FABP), was first identified in avian livers and has not been detected in mammalian species^[168–170]^. Research on FABP10 in human cancers is limited, with current studies primarily focusing on the role of FABP10 in liver cancer using transgenic zebrafish model.

#### Roles and mechanisms of FABP10 in cancer

#### FABP10 in hepatocellular carcinoma

In transgenic zebrafish models, carcinogenic gene expression (e.g., KRASV12) driven by the fabp10 promoter induces hepatocellular carcinoma (HCC). Overexpression of krasV12 activates the Ras-Raf-MEK-ERK and Wnt-β-catenin pathways, leading to hepatocyte hyperplasia and malignant transformation^[171,172]^. Studies indicate that after inhibiting the expression of carcinogenic genes, some liver cancer cells can revert to normal liver cells through FABP10-related pathways, suggesting that FABP10 may be involved in the regulation of tumor cell fate^[173]^. FABP10 regulates tumor cell lipid metabolism through fatty acid binding and transport. In PFOS (perfluorooctanesulfonic acid)-exposed liver cancer models, abnormal expression of FABP10 leads to lipid metabolism disorders, promoting tumor cell energy acquisition and proliferation^[174]^. TDCIPP (tris(1,3-dichloro-2-propyl) phosphate) activates Toll-like receptor signaling pathways, inducing hepatic inflammatory responses (e.g., neutrophil infiltration), thereby accelerating FABP10-driven liver cancer progression^[175,176]^.

#### Clinical application of FABP10

Transgenic zebrafish models constructed using the FABP10 promoter (e.g., Tg(fabp10:rtTA2s-M2;TRE2:EGFP-krasV12)) have been successfully used for antitumor drug screening. For example, pioglitazone significantly reduces liver cancer burden by inhibiting lipid accumulation^[172,177]^. Targeting FABP10 related pathways (e.g., PPARγ, NF-κB) can regulate the tumor microenvironment. For instance, curcumin activates the Nrf2-Keap1 antioxidant pathway, alleviating ethanol-induced liver injury and indirectly inhibiting liver cancer development^[178]^. Vitamin A regulates FABP10 to improve metabolic disorders, suggesting its potential as a metabolic intervention target^[179]^. The functions of FABP10 vary across species and tissues. For example, fabp10a and fabp10b in zebrafish show different expression patterns in embryos and adults, indicating the complexity of functional differentiation^[180]^. The role of FABP10 based transgenic models in precisely regulating the interplay between lipid metabolism and inflammatory pathways in human cancers requires further investigation.

### FABP11

It has been confirmed that FABP11 is identical to FABP3^[52]^.

### FABP12 (prostatic type)

#### Overview of FABP12

FABP12, also known as prostate-type fatty acid-binding protein, is a new member of the FABP family with a molecular weight of approximately 15 kDa. Its gene is located in the 8q21.13 region of human chromosome 8, which is a region frequently amplified in human prostate cancer, and encodes 140 amino acids. The *FABP12* gene evolved through tandem duplication and is conserved in mammals^[181]^. Recent studies indicate that FABP12 plays an important role in cancer progression, particularly in metastasis and chemoresistance.

#### Roles and mechanisms of FABP12 in cancer

##### FABP12 in prostate cancer

FABP12 promotes migration and invasion of prostate cancer (PCa) cells by activating the PPARγ signaling pathway, inducing epithelial-mesenchymal transition (EMT). This mechanism is associated with lipid metabolic reprogramming, characterized by increased fatty acid uptake and ATP generation dependent on β-oxidation, thereby supporting a metastatic phenotype^[182]^. FABP12 upregulates Slug (a master regulator of EMT) and apoptosis inhibitor Survivin, inhibiting docetaxel-induced apoptosis. Knockout of Slug downregulates Survivin and restores chemosensitivity in PCa cells, indicating that the FABP12-Slug-Survivin axis is a key mechanism of drug resistance^[183]^. Clinical data analysis shows that high expression of FABP12 is significantly associated with poor prognosis in prostate cancer patients^[182,183]^. Co-amplification of FABP12 with FABP5 suggests synergistic lipid metabolic pathway activation, which may become a target for combination therapy^[156]^.

##### FABP12 in hepatocellular carcinoma

In hepatocellular carcinoma (HCC), FABP12 forms a gene cluster with FABP4, FABP5, FABP8, and FABP9, which is frequently co-amplified. Although research primarily focuses on the oncogenic role of FABP5, amplification of FABP12 suggests its potential involvement in HCC progression through synergistic regulation of lipid metabolism or signaling pathways^[156]^. Specific mechanisms require further functional validation.

The oncogenic mechanisms driven by FABP12 include: PPARγ activation: regulating lipid metabolic reprogramming to support energy supply^[182]^. Slug-Survivin axis: inhibiting apoptosis and enhancing EMT^[183]^. Gene co-amplification: synergistically amplifying pro-cancer signals with other FABP members^[156]^.

#### Clinical Application of FABP12

Targeting FABP12 may become a new strategy to improve chemotherapeutic efficacy and inhibit metastasis. For example, inhibiting FABP12 or blocking its interaction with PPARγ/Slug may reverse drug resistance and delay tumor progression^[182,183]^. Future research should explore the role of FABP12 in other cancers (e.g., colorectal and breast cancer) and develop specific inhibitors. FABP12 plays a central role in prostate cancer metastasis and chemo-resistance by regulating lipid metabolism, EMT, and apoptosis-resistance, and may also participate in carcinogenesis through gene amplification in liver cancer. The elucidation of its molecular mechanisms provides new targets for developing precision therapy strategies.

## Discussion

This review goes beyond prior studies by systematically integrating molecular mechanisms, clinical implications, and cross-cancer comparisons for the FABP family. Whereas earlier reviews have focused exclusively on the structural biology or isolated roles of specific FABP isoforms^[9,10]^, this work provides a comprehensive and updated synthesis across the entire FABP family. In doing so, it highlights their diverse roles in therapeutic resistance, immunomodulation, and ferroptosis across a range of malignancies. By addressing these gaps, this review offers a roadmap for future precision oncology studies aimed at targeting FABPs in a context‑dependent manner.

In recent years, substantial advances have been made in understanding the roles of FABPs in lipid metabolism. Through their conserved β-barrel structure, FABPs bind long-chain fatty acids with high affinity, enabling efficient intracellular trafficking and dynamic metabolic responses. Specific isoforms, such as FABP1 in hepatocytes and FABP4 in adipocytes, facilitate distinct lipid metabolic pathways, including mitochondrial β-oxidation and lipid droplet formation. Beyond their classical metabolic roles, FABPs also modulate lipid-derived signaling molecules—such as eicosanoids and retinoic acids—implicating them in inflammation and cell fate determination. Notably, these metabolic regulatory functions of FABPs are increasingly recognized as central to tumor biology.

**Figure 1.**
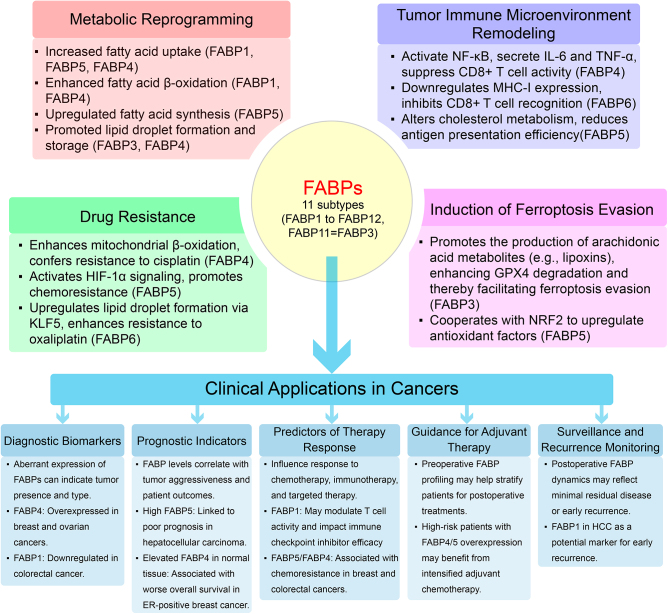
Summary of mechanisms for FABPs in cancer and their potential clinical applications.

The involvement of FABPs in cancer biology has become a rapidly expanding research field, driven by the recognition that lipid metabolism reprogramming is a hallmark of cancer. Recent studies have shown that FABPs are upregulated in a variety of tumor types and contribute to cancer initiation, progression, metastasis, and resistance to therapy. The oncogenic roles of FABPs are multifaceted: they enhance fatty acid uptake and de novo lipogenesis to meet the energy and structural demands of proliferating tumor cells; they modulate the immune microenvironment by influencing macrophage polarization and T cell infiltration; and they facilitate chemoresistance by altering apoptotic thresholds and oxidative stress responses. In addition, specific FABP subtypes exhibit tissue- and context-dependent functions, with FABP4, FABP5, FABP6, and FABP7 being the most extensively studied in breast, liver, colorectal, and brain cancers. However, emerging evidence also supports the relevance of less characterized subtypes (e.g., FABP2, FABP8, FABP12) in tumor biology. These findings have sparked interest in developing FABP-based diagnostic biomarkers and targeted therapies, although clinical translation remains limited by isoform specificity, tissue toxicity, and intertumoral heterogeneity. Figure 1 summarizes the mechanisms by which FABPs function in cancer and their potential clinical applications.

### The central hub of lipid metabolism in cancer progression

FABPs, as intracellular lipid chaperones, play a central role in cancer development by regulating lipid metabolic reprogramming, tumor microenvironment, and therapy resistance. By binding and transporting fatty acids and lipophilic molecules, FABPs directly impact energy metabolism, signal transduction, and gene expression^[9]^. In tumor cells, aberrantly activated FABPs reshape metabolic pathways to meet the high proliferative demands of cancer cells. For example, FABP1 enhances mitochondrial β-oxidation in hepatocellular carcinoma to provide energy^[18]^, while FABP4 promotes breast cancer cells to take up fatty acids from adipocytes and activates the STAT3 pathway to drive epithelial-mesenchymal transition (EMT)^[70]^. This metabolic reprogramming not only supports the energy requirements of tumor cells but also enhances their survival and invasive capabilities by regulating the activities of enzymes involved in lipid droplet formation, fatty acid oxidation, and synthesis (e.g., FASN, CPT1A)^[20,33]^.

The regulation of the tumor immune microenvironment by FABPs represents another critical dimension of their pro-tumorigenic effects. For instance, FABP4 + lipid-associated macrophages (LAMs) secrete IL-6 and TGF-β to suppress CD8^+^ T cell activity, creating an immunosuppressive microenvironment^[72]^. FABP6 downregulates MHC-I molecules expression and reduces CD8^+^ T cell infiltration, thereby promoting immune evasion in colorectal cancer^[26,128]^. This immune regulatory function synergizes with metabolic reprogramming; for example, FABP4-mediated fatty acid β-oxidation enhances the M2 polarization of tumor-associated macrophages (TAMs), further inhibiting anti-tumor immune responses^[79]^. Moreover, the role of FABPs in obesity-related cancers is particularly significant. For example, FABP4 upregulates ANGPTL4 in obesity-associated triple-negative breast cancer, promoting angiogenesis and chemoresistance^[71]^. This highlights the interplay between lipid metabolic dysregulation and the obesity-associated microenvironment as a key mechanism of FABPs-driven tumorigenesis.

Therapy resistance poses a significant challenge in cancer clinical management, and FABPs play a pivotal role in this process. For example, FABP4 enhances mitochondrial β-oxidation to reduce cisplatin-induced apoptosis in ovarian cancer^[73]^, while FABP5 promotes chemoresistance in hepatocellular carcinoma through the HIF-1α pathway^[96]^, and FABP6 enhances CRC resistance to oxaliplatin through KLF5-dependent transcription^[131]^. These mechanisms indicate that FABPs can enhance therapy resistance in tumor cells by regulating apoptosis resistance, DNA repair, and metabolic adaptability, making them important targets for the development of novel combination therapies.

### Functional heterogeneity of the FABP family and diversity of molecular mechanisms

Despite sharing the core function of lipid binding, FABPs exhibit significant functional heterogeneity across different cancer types and cell contexts. This heterogeneity is first reflected in tissue distribution and isoform specificity: FABP1 is primarily expressed in hepatocytes and promotes fatty acid oxidation in TAMs in HCC through the PPARG/CD36 axis^[18]^; FABP7, as a brain-type FABP, drives the migration and radioresistance of glioblastoma stem cells in glioblastoma (GBM) through the RXRα signaling pathway^[23]^, while in breast cancer brain metastasis, it mediates a glycolytic phenotype to adapt to the brain microenvironment^[141]^. Even the same isoform may play opposing roles in different cancers. For example, FABP5 is highly expressed and promotes tumor proliferation in hepatocellular carcinoma^[96]^, but its low expression suppresses tumor progression in colorectal cancer, a mechanism involving mTOR-mediated autophagy activation^[100]^. The paradoxical role of FABP5 across different cancers may be influenced by tumor microenvironmental context. In HCC, a lipid-rich and hypoxic microenvironment may drive FABP5-mediated lipogenesis and immune evasion, supporting its pro-tumorigenic role. In contrast, the highly immunogenic and microbially influenced colorectal environment appears to shift FABP5 activity towards mTOR-mediated autophagy and anti-tumor effects. Such differences may be attributed to the complex and heterogeneous nature of the tumor microenvironment, which can markedly influence the functional behavior of the same FABP isoform across different cancer types. Variations in stromal cell composition, the availability and type of metabolic substrates (e.g., fatty acids, glucose, amino acids), and the activation state of key signaling pathways such as PI3K/AKT, Wnt/β-catenin, or NF-κB can all contribute to these divergent roles^[20,95,184,185]^. For instance, in hepatocellular carcinoma, a lipid-rich microenvironment and hypoxic conditions may favor FABP5-mediated metabolic reprogramming and immune suppression^[96]^. In contrast, the colorectal tumor milieu—characterized by high immune cell infiltration and distinct microbial and metabolic profiles—may shift FABP5’s function toward tumor-suppressive pathways, such as mTOR-dependent autophagy^[100]^. Retrospective clinical studies showed that tissue expression levels of FABP5 are associated with prognosis in HCC patients^[96,186,187]^; however, no significant differences in serum FABP5 levels were observed between HCC patients and non-cancerous hepatitis patients^[188]^. These context-specific influences underscore the necessity of interpreting FABP function within the broader framework of tumor-specific cellular, metabolic, and immunological interactions. Notably, much of the current evidence is derived from in vitro studies and retrospective analyses, with limited validation from prospective clinical cohorts or mechanistic in vivo models. Therefore, further well-designed studies are warranted to clarify the context-dependent roles of FABPs across cancer types. To further highlight the context-dependent roles and mechanistic differences of FABP isoforms across various cancer types, we summarize these findings in Table 2, providing a cross-subtype comparison that captures both their common and distinct characteristics.

**Table 2:** Cross-Subtype Comparison of FABP Roles in Cancer

FABP name	Cancer Type(s)	Role	Key Pathway(s)	Tumor Microenvironment Context
FABP1	HCC, CRC, GC	Oncogenic/HCC	PPARG/CD36/CPT1A	Lipid-rich, hypoxic microenvironment (HCC) vs immunogenic, microbially influenced microenvironment (CRC)
		Anti-tumor/CRC		
FABP2	CRC, GC, BC	Context-dependent	Wnt/β-catenin, PPARG, BMP4	Influenced by microbiota and dietary lipids
FABP3/FABP11	NSCLC, BC, CRC, ESCC	Oncogenic	PPAR, HIF-1α	Obesity, hypoxia-associated microenvironment
FABP4	BC, OC, PC, CRC	Oncogenic	CD36, PPARG, HIF-1α	Lipid-rich microenvironment, insulin resistance
FABP5	HCC, CRC, BC, Melanoma	Context-dependent	NF-κB, HIF-1α, mTOR, PPARG	Modulated by microbiota, bile acids, and immunity
FABP6	HCC, CRC, ESCC, BC	Oncogenic	IGF, KLF5, MHC-I	Influenced by bile acids, microbiota
FABP7	GBM, BC, Melanoma, CRC	Context-dependent	RXRα, Wnt/β-catenin, MEK/ERK	Fat-rich environments, therapy resistance
FABP8	HCC, Melanoma, OC, Schwannoma	Oncogenic	SOX10, PI3K/Akt	Neurogenic microenvironment
FABP9	PCa, BC, ESCC, HCC	Oncogenic	EMT, H3K4me3, TP53	Hormone receptor activity, immune microenvironment
FABP12	PCa, HCC	Oncogenic	PPARG, Slug, Survivi n	High-fat microenvironment, chemoresistance

The diversity of molecular mechanisms further highlights the complexity of FABP functions. In terms of signaling pathways, FABPs can act through multiple pathways such as PPARγ, NF-κB, HIF-1α, and Wnt/β-catenin: FABP4 promotes lipid droplet formation in adipocytes through PPARγ^[19]^, FABP5 induces EMT and lymphangiogenesis in cervical cancer through NF-κB^[102]^, and FABP6 promotes CRC cell proliferation through the IGF signaling pathway^[129]^. The cross-activation of these pathways not only regulates metabolic reprogramming but also influences cell cycle progression, apoptosis, and epigenetic modifications. For example, FABP7 in GBM epigenetically reprograms to suppress immune-related gene expression while upregulating ferroptosis-resistance gene BMAL1, helping tumor cells evade immune clearance and ferroptosis^[138]^, which demonstrates deep integration of metabolic regulation and epigenetics.

Cell-to-cell communication within the tumor microenvironment is a critical context for FABP function. For example, breast cancer-associated fibroblasts (CAFs) promote lipid take up in tumor cells through the FABP2/FABP3 pathway^[43]^, and ovarian cancer cells uptake fatty acids released by adipocytes through FABP4 to support metastasis^[29]^. This metabolic coupling relies on FABP-mediated transcellular lipid transport, creating a “metabolic symbiosis.” Additionally, FABPs play a crucial role in interactions between immune cells and tumor cells. For example, FABP4^+^ LAMs release pro-inflammatory factors through lipolysis to suppress T cell function^[24]^, and FABP6^+^ tumor cells downregulate MHC-I molecules to reduce T cell recognition^[26]^, demonstrating the central role of FABPs in metabolic crosstalk between tumors and immune cells.

### Potential clinical applications of FABPs in surgical oncology

In the context of surgical oncology, FABPs show promise not only as diagnostic or prognostic biomarkers but also as pivotal molecular tools to assist in postoperative treatment stratification and personalized therapeutic planning. For example, preoperative profiling of FABP expression levels may guide adjuvant therapy selection, enabling surgeons and oncologists to tailor chemotherapeutic regimens based on individual tumor biology. In colorectal and breast cancers, overexpression of FABP4 and FABP5 has been correlated with recurrence risk and chemoresistance^[76,110,189–191]^, suggesting their utility in identifying high-risk patients who may benefit from intensified adjuvant treatment or early postoperative monitoring strategies. Furthermore, postoperative FABP expression dynamics could serve as molecular indicators of residual tumor burden or early recurrence, thus informing decisions on the timing and intensity of follow-up interventions. For instance, studies have shown that high expression of FABP1 in hepatocellular carcinoma is associated with increased tumor aggressiveness^[192]^, and elevated gene expression of FABP4 in normal tissue were associated with worse overall survival of patients with ER-positive breast cancer (*P-value* = 0.0089)^[193]^, highlighting their potential as molecular predictors of recurrence and long-term prognosis.

By bridging molecular insights with surgical interventions, FABPs offer a unique avenue to optimize perioperative oncologic management. Integrating FABP-based molecular signatures into surgical decision-making algorithms may enhance precision-medicine approaches and ultimately contribute to improved patient outcomes in the era of precision surgery.

### Challenges in clinical translation and future research directions

The potential of FABPs as diagnostic biomarkers and therapeutic targets for cancer has been widely validated, but their clinical translation faces multiple challenges. In diagnostics, while FABP1 combined with TRIB3 can improve the sensitivity of early gastric cancer diagnosis^[34]^, and FABP6 combined with CEA can enhance CRC diagnostic efficacy^[127]^, the specificity of single biomarkers and expression heterogeneity across different cancer types limit clinical application. Future efforts should integrate multi-omics approaches (e.g., transcriptomics, metabolomics, and single-cell sequencing) to identify panels of FABP-related molecular biomarkers. Combined with tumor staging, subtyping, and microenvironment characteristics, precise diagnostic and prognostic models can be established.

In terms of therapy, strategies targeting FABPs include small-molecule inhibitors, combination therapies, and metabolic interventions. For example, Orlistat inhibits FABP1 activity to enhance immunotherapy efficacy in HCC^[18]^, BMS309403 targets FABP4 to inhibit ovarian cancer metastasis and enhance chemosensitivity^[29]^, and SBFI-26 induces ferroptosis in liver cancer cells by inhibiting FABP5^[99]^. However, existing inhibitors face issues of insufficient specificity (e.g., Orlistat also inhibits lipase) and in vivo stability, which urgently needs to be addressed. Moreover, the physiological functions of FABPs in normal tissues (e.g., FABP4’s role in adipocyte energy homeostasis) may lead to off-target toxicity. Future research should develop highly selective inhibitors based on structural differences in FABPs (e.g., specificity of ligand binding pockets) and explore tissue-targeted delivery systems (e.g., nanoparticle carriers) to minimize side effects.

In-depth mechanistic studies are key to breaking through translational bottlenecks. Current understanding of FABPs’ roles in cancer stem cells (CSCs) and metastatic colonization remains unclear. For example, how FABP7 regulates the metabolic phenotype of GBM stem cells^[139]^ and how FABP4 affects the maintenance of breast cancer stem cells^[72]^. Answering these questions will provide new perspectives for targeting CSCs. Additionally, the interplay between FABPs and emerging metabolic pathways (e.g., cuproptosis, ferroptosis) deserves further investigation. For instance, FABP3 resists ferroptosis by inhibiting arachidonic acid metabolism^[21]^, and FABP5 inhibitors induce ferroptosis by promoting lipid peroxidation^[99]^, revealing FABPs’ critical roles in regulating cell death modalities.

From a methodological perspective, single-cell sequencing and spatial transcriptomics can parse the cell-specific expression of FABPs within the tumor microenvironment (e.g., high expression of FABP4/5 in TAMs and fibroblasts). Combined with gene-editing models (e.g., CRISPR screening), key downstream targets and synthetic lethal partners of FABPs can be identified. In clinical applications, stratified treatment strategies based on FABP expression profiles (e.g., prioritizing CD36 antibodies combined with PD-1 inhibitors for patients with high FABP4 expression) may improve treatment response rates and require validation through large-scale clinical trials. At the same time, it is vital to consider and mitigate the off‑target effects associated with FABP inhibition, especially those affecting adipocyte insulin sensitivity and systemic lipid metabolism. To overcome these limitations, emerging therapeutic platforms such as targeted nanoparticle delivery and protein degraders such as proteolysis-targeting chimera (PROTAC) are gaining attention as promising strategies for achieving precision targeting while minimizing adverse effects.

### Limitations

Despite providing a comprehensive overview of FABPs in cancer biology, this review has several limitations. First, most of the available evidence is derived from in vitro and in vivo preclinical studies, with relatively limited clinical trial data supporting the translational application of FABPs as biomarkers or therapeutic targets. This gap underscores the need for more well-designed clinical investigations to validate the clinical utility of FABP-targeted strategies in cancer diagnosis, prognosis, and treatment. Second, the functional characterization of certain FABP subtypes, such as FABP10, remains insufficient due to the scarcity of relevant studies. This limits our understanding of their potential roles in tumorigenesis and its applicability in clinical oncology. Third, although we applied a systematic literature search strategy and screened for relevance, our study selection was limited to English-language studies available in PubMed and Web of Science databases, which may introduce selection bias and lead to omission of potentially relevant findings from other sources. Finally, given the rapidly evolving nature of tumor metabolism and immunology, the mechanistic insights summarized here may not fully capture the latest developments or reflect context-dependent variability across tumor subtypes and microenvironments. Additionally, interpatient and intra-tumoral heterogeneity may limit the generalizability of some FABP-based findings across cancer types. Future studies integrating multi-omics data and functional validation in patient-derived models will be crucial to deepen our understanding of FABP biology and its clinical relevance.

## Conclusion

The FABP family regulates lipid metabolism, tumor microenvironments, and therapy resistance, serving as a central link between metabolic reprogramming and cancer progression. Despite significant functional heterogeneity among isoforms, their shared characteristics—lipid-binding capacity and signaling pathway regulation—provide a unified framework for cancer diagnosis and therapy. Future research should advance mechanisms, drug development, and clinical applications in tandem to overcome specificity and efficacy challenges, ultimately achieving precision and personalized cancer therapy. Research on FABPs not only deepens the understanding of tumor metabolic dependencies but also provides a new theoretical basis for the combination of metabolism-targeted therapies and immunotherapy, potentially ushering in a new paradigm for cancer treatment.

## Data Availability

All data analyzed in this review are derived from publicly published studies, and the original data can be obtained through the supplementary materials of the corresponding papers or open databases.
